# Conium as a Local Anæsthetic

**Published:** 1888-08

**Authors:** 


Conium as a Local Anæsthetic.—Attention has been called to the value of hemlock as a local anæsthetic in painful affections of the rectum and anus by Dr. Whitla (*Practitioner*, April, p. 250). He states that he has found an ointment of conium very useful when applied in pruritus ani, especially when associated with or caused by hemorrhoids or fissures in the anus or lower part of the rectum. Having found the official extract of conium unreliable, Dr. Whitla prepares an ointment from the succus. This he does by evaporating two ounces of the succus slowly, at a temperature below 150 degrees F., until reduced to one and a-half or two drachms, and then triturating the syrupy residue with sufficient lanolin to make the weight up to one ounce. The product is a smooth, adhesive, stable ointment, of a light brown or dark fawn color.—*Pharm. Journal.*

